# Blood Changes in Disease

**Published:** 1852-11

**Authors:** 


From L'Union Medicale.

*Blood Changes, in Disease.*

A recent elaborate memoir on diseases of the blood, presented to the Academy of Sciences by MM. Becquerel and Rodier, winds up with the following conclusions :—

1. In most chronic diseases, or in consequence of various hygienic influences, the three principal elements of the blood,—viz., the red particles, the fibrine, and the albumen, may diminish or augment in quantity, separately or conjointly.

2. The red particles diminish in most prolonged chronic diseases, especially in organic diseases of the heart, in Bright's disease, chlorosis, cachexia, hæmorrhages, the last period of tuberculosis, the cancerous diathesis; the globules diminish also, when the individual has been insufficiently nourished, or has been exposed to bad hygienic conditions, such as insufficiency of air, want of light, or to humidity.

3. The albumen diminishes in the last stage of heart diseases, great symptomatic anæmia, cancerous diathesis, and when the nourishment is insufficient.

4. The fibrine remains normal, or is sometimes augmented in acute scurvy. In chronic scurvy, and in the symptomatic scorbutic state which attends some chronic maladies, especially in organic diseases of the heart, it diminishes.

5. In all the above cited cases, the water of the blood increases in amount.

6. The diminution of globules is shown especially by decoloration of the skin, palpitations, dyspnœa, systolic murmur at the base of the heart, arterial and venous murmurs.

7. The diminution of albumen produces dropsy; speaking generally, dropsy is the symptom of diminution of albumen.

8. The diminution of fibrine shows itself by cutaneous and mucous hæmorrhages.

9. In the symptomatic anæmia of great hæmorrhages, of bad nourishment, and of too great catamenial flow, the specific gravity of the blood diminishes, the water augments, the blood globules diminish, the albumen remains normal or slightly diminishes; the fibrine remains the same.

10. In chlorosis, which is an entirely different disease from anæmia, the blood may be completely normal. When any changes occur, they are in the augmentation of the water, the diminution of the red particles, and the conservation of its normal figure, or the augmentation of the fibrin.

11. In acute Bright's disease, the fibrin remains the same, the albumen diminishes; in chronic Bright's disease, the globules and albumen diminish; the fibrine remains normal or diminishes.

12. Most dropsies, called essential, are due to a diminution of the albumen.

13. In heart disease, the blood deteriorates more and more, as the disease advances. The changes consist in simultaneous diminution of the red globules, albumen, and fibrine.

14. In acute scurvy, the blood is not altered; in chronic scurvy, the fibrine is diminished, and the globules augmented.

15. Quinine and generous diet is the best treatment for diminution of the albumen; vegetable acids and good diet for diminution of the fibrine; iron for the diminution of the red particles.

